# Supplementary material for: Analysis of 13,312 benthic invertebrate samples from German streams reveals minor deviations in ecological status class between abundance and presence/absence data
Source: PLoS One. 2019 Dec 23;14(12):e0226547. doi: 10.1371/journal.pone.0226547 (PMC6927632; doi:10.1371/journal.pone.0226547)
Supplement: S1 Fig — (PDF) [file pone.0226547.s001.pdf]

**Analysis of 13,312 benthic invertebrate samples from German streams reveals minor deviations in ecological status class between abundance and presence/absence data**

Dominik Buchner[1§*], Arne J. Beermann[1,2], Alex Laini[3], Peter Rolauffs[4], Simon Vitecek[5,6], Daniel Hering[2,4], Florian Leese[1,2§*]

# Supplementary Figure S1

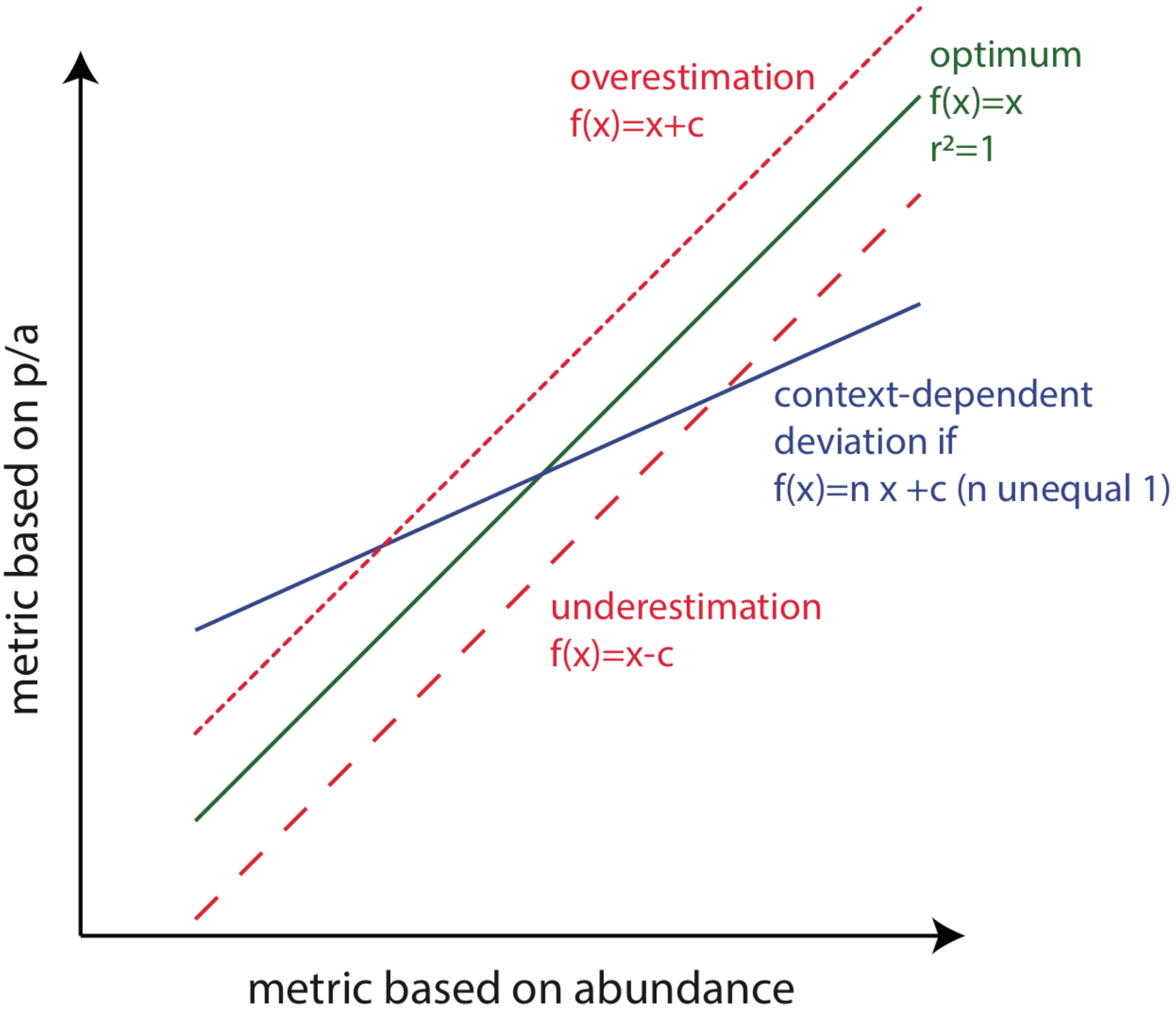


**Figure S1:** Expected relationships between abundance (*x*-axis) and presence/absence-based (*y*-axis) metrics.
