# Supplementary material for: Analysis of 13,312 benthic invertebrate samples from German streams reveals minor deviations in ecological status class between abundance and presence/absence data
Source: PLoS One. 2019 Dec 23;14(12):e0226547. doi: 10.1371/journal.pone.0226547 (PMC6927632; doi:10.1371/journal.pone.0226547)
Supplement: S2 Fig — Deviation of the observed classification in the ecological status class (y-axis) in relation to the contribution of abundance information for the calculation of the module (CAB). (PDF) [file pone.0226547.s002.pdf]

Analysis of 13, 13,312 benthic invertebrate samples from German streams reveals minor deviations in ecological status class between abundance and presence/absence data

Dominik Buchner[1§*], Arne J. Beermann[1,2], Alex Laini[3], Peter Rolauffs[4], Simon Vitecek[5,6], Daniel Hering[2,4], Florian Leese[1,2§*]

Supplementary Figure S2

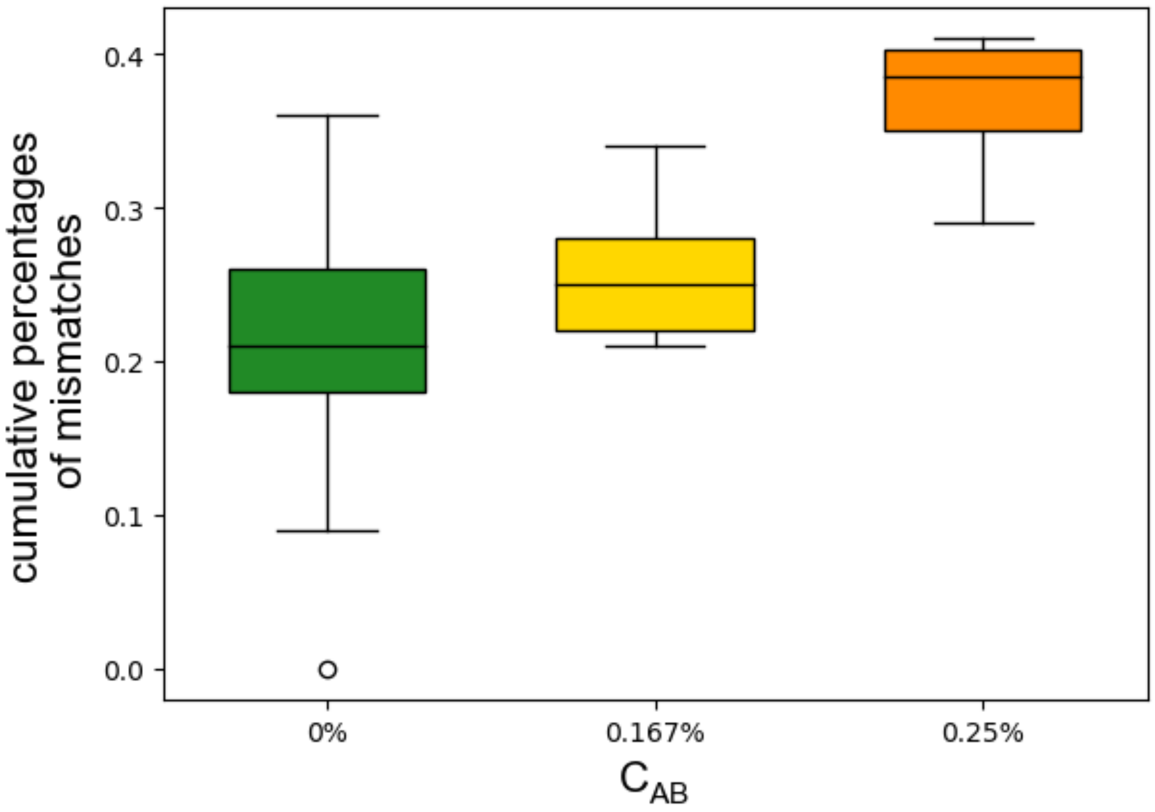

Figure S2: Observed cumulative percentages of mismatches (y-axis) between the three different levels of C_{AB} (x-axis).
