# Supplementary material for: Analysis of 13,312 benthic invertebrate samples from German streams reveals minor deviations in ecological status class between abundance and presence/absence data
Source: PLoS One. 2019 Dec 23;14(12):e0226547. doi: 10.1371/journal.pone.0226547 (PMC6927632; doi:10.1371/journal.pone.0226547)
Supplement: S3 Fig — Comparison of abundance (‘abd’, black line with green confidence interval) and presence/absence (red dots) assessment results for 627 stream sites for which the transformation led to a status class shift from 2 to 3. Background shading indicates ecological status class intervals. These are geometrically defined for the %EPT and German fauna index (GFI) and therefore much narrower than for the German saprobic index. (PDF) [file pone.0226547.s003.pdf]

Analysis of 13,312 benthic invertebrate samples from German streams reveals minor deviations in ecological status class between abundance and presence/absence data

Dominik Buchner[1§*], Arne J. Beermann[1,2], Alex Laini[3], Peter Rolauffs[4], Simon Vitecek[5,6], Daniel Hering[2,4], Florian Leese[1,2§*]

Supplementary Figure S3

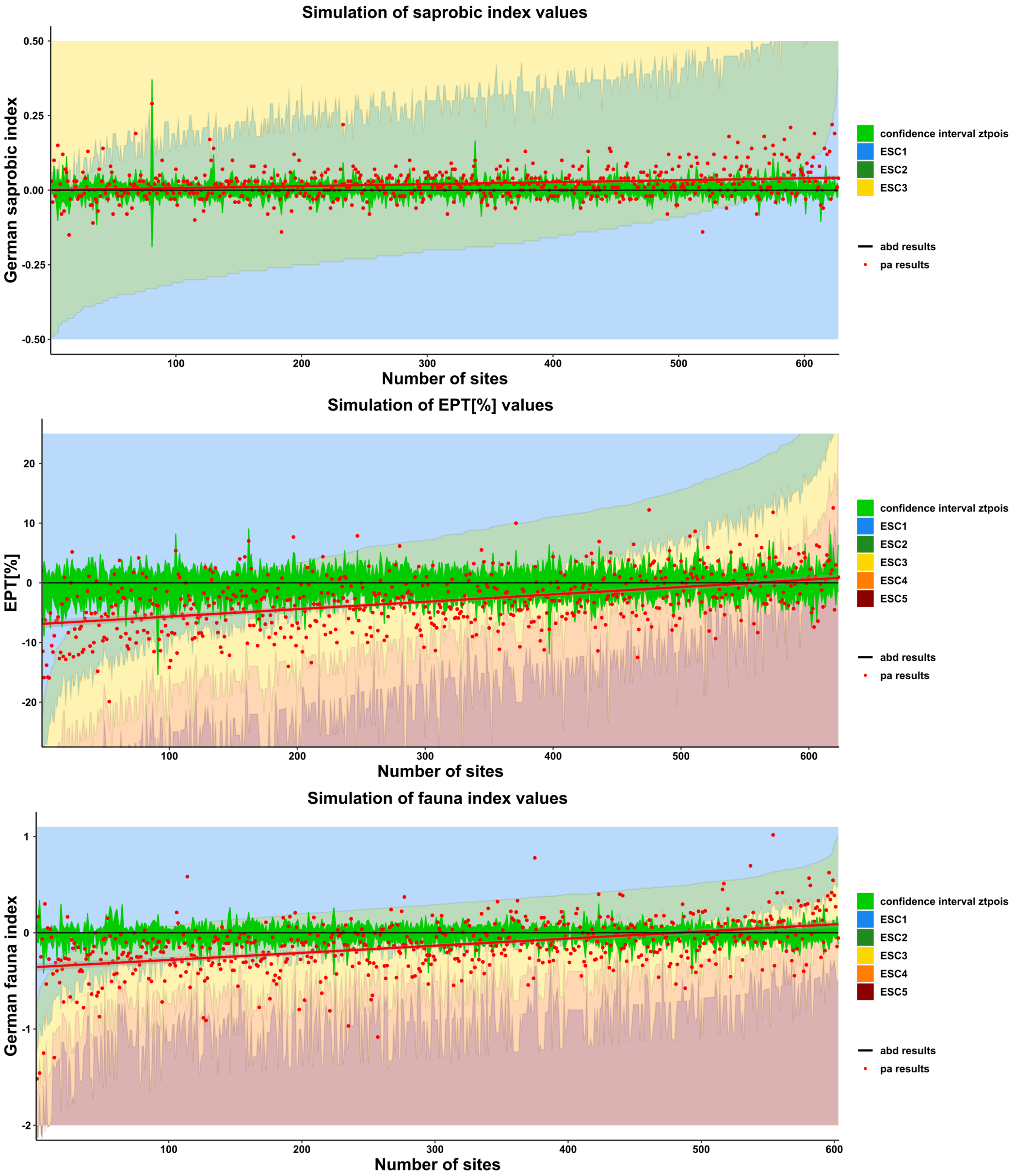

Figure S3: Comparison of abundance ('abd', black line with green confidence interval) and presence/absence (red dots and red line with a linear model fitted to the red dots to indicate trends) assessment results for 627 stream sites for which the transformation led to a status class shift from 2 to 3. Background shading indicates ecological status class intervals. These are geometrically defined for the %EPT and German fauna index and therefore much narrower than for the German saprobic index.
